# pRb2/p130 protein expression and *RBL2 *mutation analysis in Burkitt lymphoma from Uganda

**DOI:** 10.1186/1472-6890-9-6

**Published:** 2009-08-19

**Authors:** Sam Kalungi, Solrun J Steine, Henry Wabinga, Leif Bostad, Anders Molven

**Affiliations:** 1 Section for Pathology, the Gade Institute, University of Bergen, Bergen, Norway; 2 Department of Pathology, Makerere University College of Health Sciences, Kampala, Uganda; 3 Department of Pathology, Haukeland University Hospital, Bergen, Norway

## Abstract

**Background:**

The members of the retinoblastoma protein family, pRb, p107 and pRb2 (p130), are central players in controlling the cell cycle. Whereas disturbed function of pRb is commonly seen in human cancers, it is still an open question whether pRb2 is involved in tumorigenic processes. However, altered subcellular localization of pRb2 and mutations in the pRb2-encoding gene *RBL2 *have been described for some tumours, including Burkitt lymphomas (BL).

**Methods:**

We retrieved 51 biopsy specimens of endemic BL cases from Uganda. The expression of pRb2 was determined by immunohistochemistry. Exons 19-22 of the *RBL2 *gene, the region known to contain a nuclear localization signal, were screened for mutations by PCR amplification and direct DNA sequencing.

**Results:**

Nearly all of our cases (84.0%) were positive for pRb2 protein expression although this protein is a marker for growth arrest and Burkitt lymphoma is characterized by a high proliferation rate. Of the positive cases, 73.8% were scored as expressing the protein at a high level. Subcellular pRb2 localization was predominantly nuclear and no cases with expression restricted to the cytoplasm were observed. We did not detect any *RBL2 *mutations in the part of the gene that encodes the C-terminal end of the protein.

**Conclusion:**

The majority of endemic BL cases from Uganda express pRb2, but somatic *RBL2 *mutations affecting the protein's nuclear localization signal appear to be rare.

## Background

The retinoblastoma protein pRb and its relatives p107 and pRb2 (p130) constitute a family of proteins sharing common structural organization and significant degree of sequence similarity. These proteins have critical functions in the control of cell proliferation and differentiation [1,2]. Each of the three proteins can elicit G1 growth arrest by binding to and inhibiting the E2F transcription factors. The retinoblastoma (Rb) family of proteins exhibit their growth-suppressive properties in a manner which is both cell type- and cell cycle-dependent. Hence, they are not functionally redundant although they may complement each other [2-4].

The normal functions of the Rb protein family may be down-regulated or completely ablated by a variety of mechanisms including mutations [1,5]. Deletions and other alterations of the genes that participate in the regulation of Rb proteins, such as *CDKN2A*, *CDK4 *and *CCND1*, occur frequently in many tumours [6,7]. This illustrates that disruption of the signaling pathway in which pRb, p107 and pRb2 act, is a common event in tumorigenesis.

Among the members of the Rb protein family, pRb2 is the most abundant in G0. It maintains G0 arrest in quiescent or differentiated cells, controls the transition from G1 to S phase, and is a key regulator of growth arrest in cellular senescence [8]. The *RBL2 *gene, which encodes the pRb2 protein, contains 22 exons [9] and has been mapped to human chromosome 16q12.2 [10]. The pocket domain of pRb2 is encoded by exons 10-13 (domain A) and 17-20 (domain B). The term 'pocket' relates to the conserved domain through which pRb, p107 and pRb2 bind viral oncoproteins and cellular transcription factors of the E2F family [1]. Notably, the pRb2 protein depends upon correct subcellular localization and one or more nuclear localization signals (NLS) in order to achieve full biological activity [11].

Expression of pRb2 has been demonstrated in a variety of normal tissues and tumours, for example in hematopoietic and epithelial cells [12], atypical endometrial hyperplasia and carcinoma [13] and non-Hodgkin lymphomas [14]. The protein has been reported to be involved in the pathogenesis and progression of vulvar squamous cell carcinomas [15] and salivary gland tumours [16] and may predict prognosis in choroidal melanomas [17]. The role of pRb2 in cancer development is, however, still debatable [18]. For instance, pRb2 is not of prognostic significance in cervical intraepithelial neoplasia [19]. Moreover, loss of this protein may not be sufficient to deregulate the orderly progression of the cell cycle, as mice unable to express pRb2 do not exhibit any altered tumour predisposition or developmental defects [3].

The pRb2-containing genomic region, i.e. the long arm of chromosome 16, is often lost in carcinomas of the breast, prostate, liver, and ovary [20-23]. Somatic mutations in the *RBL2 *gene have, however, rarely been reported. The few examples include a small cell lung cancer cell line [24] and nasopharyngeal carcinoma from Northern Africa [25]. There are conflicting findings in Burkitt lymphomas (BL) where *RBL2 *mutations were observed in 11 of 13 endemic African cases, whereas AIDS-related BL showed no mutations [26]. In a series of sporadic BL from Brazil no mutations in the *RBL2 *gene were found [27].

The aim of this study was to investigate pRb2 expression in endemic BL cases from Uganda and to search for tumour-associated, somatic *RBL2 *mutations that could be involved in deregulated cell cycle control.

## Methods

### Selection of cases and conventional histology

Formalin-fixed paraffin-embedded tissue blocks from cases of BL were obtained from the archives of the Department of Pathology, College of Health Sciences, Makerere University, Kampala, Uganda. The sections were re-embedded at the Department of Pathology, the Gade Institute, Haukeland University Hospital. Before pathology re-investigation was done, all specimens were delinked from personal identifiers. The cases were revised by two pathologists (SK, LB) to confirm the histological diagnosis of BL. The final diagnosis was based on a combination of characteristic histology and immunophenotype (CD45+, CD20+, CD10+, BCL6+, BCL2- and Ki-67 > 95%). Information on age, sex and site of biopsy was obtained when available. Permission to perform the study including accessing the lymphoma samples was obtained from the Pathology Department, Makerere University, Faculty of Medicine (now College of Health Sciences), Kampala, Uganda. Further ethical approval and material transfer agreement were not compulsory at the time the study was initiated. The Norwegian part of the study was covered by a project approval by the University of Bergen and by the Department of Pathology, Haukeland University Hospital's general permission to investigate material from human beings. The study was carried out according to the Helsinki Declaration.

### Immunohistochemistry

Five-μm sections of the tissue blocks were cut and placed on sialinized microscope slides. Immunostaining was performed using the automated immunohistochemical staining system TechMate (Dako, Glostrup, Denmark) after heat-induced epitope retrieval. The sections were first deparaffinized and hydrated through a graded series of alcohol and water. The antigen retrieval procedure involved the use of a microwave oven for pre-heating the slides in retrieval buffer (10 mM Tris, pH 9.0; 1 mM EDTA) for 10 minutes until boiling, then heating for additional 15 minutes. Endogenous peroxidase activity was blocked by treatment with hydrogen peroxide S2001 (Dako) for 5 minutes. The slides were then incubated for 60 minutes with an Rb2/p130 monoclonal antibody diluted 1:100 (clone 130P215 from Abcam, Cambridge, U.K). The antibody clone 130P215 was raised against a peptide corresponding to amino acids 878-913 of human pRb2, thereby recognizing a region near the C-terminus but different from the region screened in the mutational analysis. Detection of antibody binding was performed using the Envision HRP kit K4061 (Dako). Heamatoxylin was used as a counter stain. The pRb2 staining pattern was evaluated as high (more than 50% of the tumour cells staining), low (1%-50% of tumour cells staining), or absent (no staining). A tissue micro array (TMA) block was made from 1-mm cylinders punched out from a representative tumour area of each BL case. As validation of the staining interpretation, whole sections from ten of the TMA cases were cut, treated and evaluated in the same way as the TMA block.

### DNA extraction from tissue sections

Depending on the size of the tumour tissue, two or three 8-μm sections were cut. The sections were placed in 190 μl buffer G2 and 10 μl proteinase K solution of the MagAttract DNA mini M 48 kit (Qiagen, Hilden, Germany) and dissolved by shaking overnight at 56°C. DNA was then purified by using this kit in combination with a GenoM48 BioRobot system (Qiagen) in accordance with the manufacturer's instructions. All 14 cases selected for the molecular genetic analysis were regarded as classical BL.

### PCR and DNA sequencing

Exons 19-22 were PCR-amplified (exons 19, 20 and 22 as two overlapping fragments) and screened for mutations by direct sequencing. Primer sequences are given in Table 1. The exon 21 product did not fully cover the exon, but included all regions where *RBL2 *mutations have been reported previously [26]. In general, PCR reactions were run in a total volume of 25 μl with 0.3 μM of each primer using the AmpliTaq Gold PCR Mastermix (Applied Biosystems, Foster City, CA, USA) or Taq DNA polymerase with Q-solution (Qiagen). Samples were subjected to initial denaturation at 95°C for 15 min, 45-50 cycles at 95°C for 50 s, annealing (see Table 1 for temperatures) for 50 s, and elongation at 72°C for 1 min, followed by a final elongation step at 72°C for 7 min. PCR products were purified by using enzymatic treatment with ExoSAP-IT (USB, Cleveland, OH, USA). The sequencing primers were identical to the PCR primers and the PCR products were sequenced in both directions employing the BigDye Terminator Cycle Sequencing kit, Version 1.1 (Applied Biosystems). The sequence reactions were analyzed on an ABI Prism 3100 Genetic Analyzer with Sequencing Analysis software, Version 3.7 (Applied Biosystems).

**Table 1:** Primers used for amplification and sequencing of the *RBL2 *gene.

Exon		Primer sequence (5'-3')	Annealing temperature	Product size (bp)
19-1	Forward	AGGGTGTGCTCAACAAATAC	50	142
	Reverse	CTGTTGGAGAATTCTGATGG		
19-2	Forward	TCTCTAAAGGTGTATAGAAGTG	50	161
	Reverse	GAAAAGCTCCTAATTTTGTC		
20-1	Forward	ATTAGCAGCCTTTGTACTGAC	55	148
	Reverse	ACTGTTGGCACCTGTGAGG		
20-2	Forward	AGCAGTGCTCCTCCCACA	50	174
	Reverse	AAAGTATGAAATAATCCCTG		
21	Forward	CTCTCTCCCTATCCATTTG	50	143
	Reverse	GGACTGTTGCTGAAGTAATAG		
22-1	Forward	CTGAGCTATGTGCATTTGCA	52	148
	Reverse	TTTGCAGGTGATTCACTTCC		
22-2	Forward	AAAGAGAGGAATTCTTTTGG	54	145
	Reverse	AGAGTTTAATACAAGAGACT		

### Statistical analysis

Pearson's chi-square test was used to evaluate any relationships between the different clinicopathological parameters. A result was considered significant when its *p*-value was < 0.05. The statistical software package SPSS, Version 15 was used.

## Results

A total of 51 BL cases were included in the study, 21 (41.2%) of whom were males and 29 (56.9%) females. The gender was unknown in one case. The mean age of the patients was 6.9 years and the median was 7 years with a range of 3-13 years. Features of the cases included in the study are listed in Table 2. Facial tumours (n = 26; 51.0%) were more abundant than abdominal tumours (n = 21; 41.2%). The site of the tumour was unknown in four (7.8%) cases. Of the facial tumours, oral tumours were most common (n = 23) with the remaining three cases arising from the orbit. Thirteen of the abdominal tumours were from the ovary, one from the testicle, one from the kidney, one from the liver and five from unspecified sites in the abdomen. Oral cavity tumours were more common among the males while abdominal tumours dominated among the females. Facial tumours were more frequent in children below eight years but this association between tumour site and patient age did not reach statistical significance (*p *= 0.08).

**Table 2:** Clinical information and pRb2 staining scores for the studied BL cases.

Case number	Age	Sex	Biopsy/tumour site	pRb2 TMA staining score*
**1**	6	F	Palate	3
2	6	F	Ovary	3
3	7	F	Buccal cavity	3
4	7	M	Gingiva	3
5	5	F	Gingiva	3
6	6	F	Ovary	3
7	6	F	Buccal cavity	1
8	7	F	Buccal cavity	2
9	7	F	Ovary	3
10	8	F	Ovary	3
11	7	M	Unknown	3
**12**	6	M	Gingiva	3
13	4	M	Unknown	3
14	5	M	Jaw	3
**15**	9	M	Abdomen	3
16	9	F	Abdomen	1
17	7	M	Gingiva	3
**18**	7	F	Kidney	2
**19**	12	F	Ovary	2
20	4	F	Gingiva	3
21	4	F	Gingiva	n.a.
**22**	13	M	Liver	3
**23**	13	F	Abdomen	3
24	7	M	Orbit	3
**25**	5	F	Ovary	3
**26**	3	F	Ovary	1
**27**	3	F	Ovary	3
**28**	5	F	Ovary	3
**29**	5	M	Orbit	3
30	n.a.	n.a.	Unknown	3
31	12	F	Abdomen	3
32	12	F	Gingiva	3
**33**	5	M	Jaw	2
34	8	F	Ovary	2
35	6	M	Abdomen	3
**36**	8	F	Jaw	3
37	5	M	Orbit	1
38	5	M	Gingiva	2
39	8	F	Ovary	2
40	5	F	Ovary	1
41	5	F	Jaw	2
42	7	F	Ovary	2
43	6	M	Gingiva	3
44	7	M	Gingiva	3
45	5	M	Testis	1
46	8	M	Gingiva	1
47	8	F	Unknown	3
48	8	M	Gingiva	1
49	6	F	Jaw	3
50	7	M	Jaw	2
51	5	M	Jaw	2

* 1 = absent (0% of tumour cells staining); 2 = low (1-50% of tumour cells staining); 3 = high (>50% of tumour cells staining). Case numbers in bold indicate those cases from which biopsy tissues were used in the mutation analysis. n.a. = not available.

**Figure 1:**
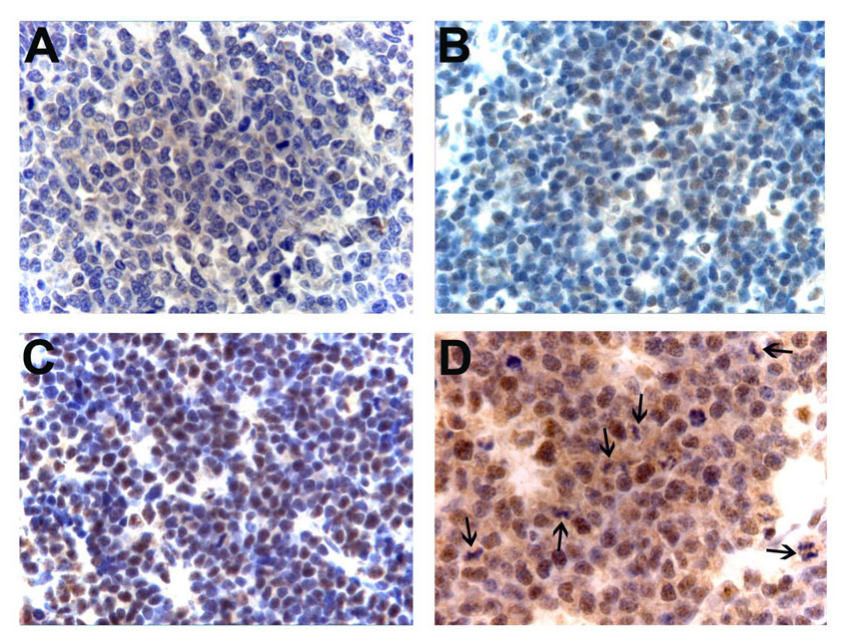
**Representative examples of pRb2 staining in BL patients from Uganda**. A) Negative staining (original magnification: ×400). B) Low staining, i.e. less than 50% of tumour cells expressing pRb2 (original magnification: ×400). C) High staining, i.e. more than 50% of tumour cells expressing pRb2 (original magnification: ×400) D) Cytoplasmic expression of pRb2 in tumour cells undergoing mitosis. Dividing cells are indicated by arrows (original magnification: ×630).

Forty-two (84.0%) of the 50 cases for which immunohistochemical data could be obtained, stained positively for pRb2 while eight (16.0%) were negative (Table 2). Among the positives, immune reactivity varied from close to 100% of the tumour cells to only a few positive cells in the section (Figure 1A-1C). The staining was predominantly nuclear. Also, cells undergoing mitosis were positive, with immune reactivity present throughout the cytoplasm (Figure 1D). Table 2 shows the staining scores for the cases included in the study. We did not find any statistically significant association between pRb2 staining (positive/negative) and age below 8 years (*p *= 0.69), or gender (*p *= 0.71), or tumour site (*p *= 1.00). Of the 42 positive cases, 31 (73.8%) cases were scored as highly expressing tumours and 11 (26.2%) as low-expressing. pRb2 staining (high versus low) did not show any statistically significant association with tumour site (*p *= 0.86), gender (*p *= 0.88), or age (*p *= 0.71).

**Figure 2:**
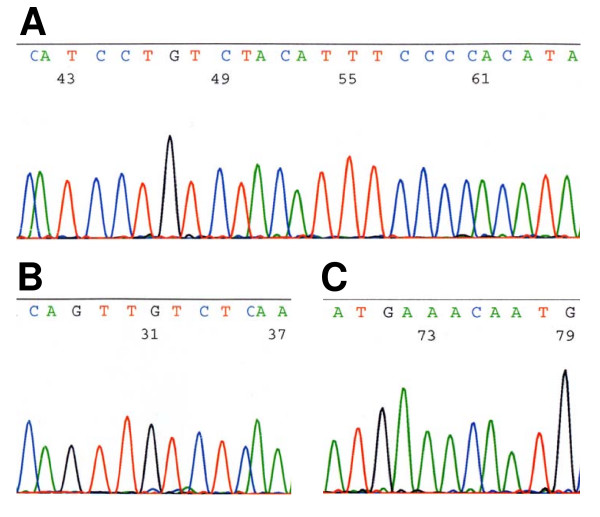
**Representative sequences in our material from the exon 21 regions in which *RBL2 *mutations were reported previously**. A) The part of exon 21 where most of the *RBL2 *mutations have been observed (ref. 26). B and C) Parts of exon 21 also previously reported to be mutated in BL.

Fourteen cases were selected for mutation analysis. The criteria for selection were classical BL histopathology, adequate amount of tissue available for repeated DNA extraction and a high ratio of lymphoid cells versus other cells in the section. Exons 19-22 whose C-terminal part encodes a pRb2 nuclear localization signal and where mutations have been reported previously, were screened. We were able to amplify and sequence 9/14 (64%) of the cases for exon 19-1, 12/14 (86%) for 19-2, 10/14 (71%) for 20-1, 8/14 (57%) for 20-2, 12/14 (86%) for exon 21, and 11/14 (79%) for exons 22-1 and 22-2. Three or more exons could be amplified in twelve of the cases, two exons in one case and one exon in one case. We did not detect *RBL2 *mutations in any of the exons. Figure 2 illustrates the DNA sequences from representative BLs in our study.

## Discussion

In the present study, we have analyzed pRb2 expression and searched for *RBL2 *mutations in endemic Burkitt lymphoma from Uganda. The median age of the patients in our material was 7 years, similar to what other reports on BL from Uganda have indicated as typical [28,29]. The number of oral tumours in our series is almost equal to that of abdominal tumours. Some studies have suggested a preponderance of oral tumours in BL, others have reported that abdominal tumours are most frequent [29-31]. Oral tumours may predominate in clinical series because they are easily seen, cause difficulty in feeding due to loosening of teeth and are disfiguring so medical attention is sought early. Oral tumours are also more accessible for biopsy compared to BL in other sites. Among males with BL, we found oral tumours to be most common, whereas in females, tumours of the ovary predominated.

The majority of tumours in our series (84.0%) were positive for pRb2 expression. In a series of untreated patients with non-Hodgkin's lymphoma (various types), low levels of pRb2 were reported to correlate with high levels of p107 and proliferation-associated proteins [32]. It may therefore seem surprising that BLs, having a proliferation rate close to 100%, express high levels of pRb2, which is considered a marker of growth arrest. The explanation could be that the pRb2 pathway is functionally impaired in BL, expression being deregulated along the cell cycle or the protein accumulating due to inhibited degradation. In this regard, it is noteworthy that high levels of pRb expression, the canonical member of the retinoblastoma protein family, has been found in high growth rate lymphomas [33].

High expression of pRb2 has also been linked to virus-associated oncogenesis [34]. Because endemic BL is associated with Epstein-Barr virus in at least 95% of the cases [30], a possible scenario could be that the pRb2 level increases in response to viral oncogenic activity. This might also explain the observation that pRb2 expression is elevated in AIDS-related lymphomas [14], although the fundamental differences between HIV and Epstein-Barr virus should be kept in mind.

The proportion of tumours with absent pRb2 expression (16.0%) in our series is comparable to what has been reported in non-lymphoid tumours [35,36]. Loss of pRb2 expression could be caused by inactivating mutations (as in other tumour suppressor genes) or by stimulated degradation. It remains to be investigated whether BLs negative for pRb2 constitute a subset of tumours with specific biological properties affecting for example patient survival.

The staining pattern in our pRb2-positive BL cases, was predominantly nuclear. In normal tissues, pRb2 expression has been reported to be confined to cell nuclei and the perinuclear membrane [12]. Concomitant nuclear and cytoplasmic staining has been observed in prostate cancer and hepatocellular carcinoma cells [35,36]. Normal human lymphocytes and osteosarcoma cell lines exhibited exclusive nuclear pRb2 localization whereas lymphoid tumour cell lines were noted to express the protein in the cytoplasm [37]. Cytoplasmic pRb2 staining was also reported in the cases of endemic BL which had *RBL2 *mutations [26]. The cytoplasmic localization of pRb2 in BL tumour tissue and in lymphoid cell lines has been attributed to mutations which lead to disruption of the NLS [26,37]. However, other authors have reported that neither deletion nor mutation of C-terminal NLS sequences resulted in complete abolishment of nuclear pRb2 localization [11]. The observed differences in level of expression and subcellular localization of pRb2 may be due to differences in the phases of the cell cycle in which the cells are studied, hyperphosphorylation of the protein which may affect NLS activity, the presence of nucleocytoplasmic protein shuttling and, of course, to different properties of the antibodies used for detection.

We did not observe any mutations in *RBL2 *in exons 19-22 in the 14 cases that we examined. Our results are therefore in contrast with those of Cinti et al. [26] who reported *RBL2 *mutations in 11 of 13 endemic BL cases from Kenya. We cannot exclude the possibility that the discrepancy is explained by inherent differences in materials from Uganda and Kenya regarding patient-specific and/or viral-specific factors. A limitation of our study is that we were not able to amplify and sequence all four exons in all cases, probably because of the varying fixation conditions (including the use of unbuffered formalin) in the African material. We nevertheless found that *RBL2 *mutations must be rarer in BL than previously suggested, a conclusion which is supported by the report of Klumb et al. [27] who were unable to detect such mutations in sporadic BL from Brazil. Moreover, none of our cases showed predominant cytoplasmic staining which could indicate the presence of mutations. Finally, we note that in the Catalogue of Somatic Mutations in Cancer (COSMIC, see ) there are no entries of somatic mutations in the *RBL2 *gene, despite recent large-scale efforts to sequence cancer cell genomes.

## Conclusion

We have shown that endemic Burkitt lymphoma from Uganda tend to express pRb2, a cell cycle protein associated with growth arrest, although intense cell proliferation is a characteristic feature of this cancer type. However, somatic mutations in the part of the *RBL2 *gene that is important for nuclear localization, appear to be rare.

## Competing interests

The authors declare that they have no competing interests.

## Authors' contributions

SK participated in the design of the study, performed and evaluated the immunohistochemistry, performed the PCR part of the mutation analysis, and wrote the paper. SJS designed the mutation analysis and performed the DNA sequencing. HW participated in the design of the study. LB re-evaluated the BL cases and participated in the design of the study and the evaluation of the immunohistochemistry. AM participated in the design of the study and wrote the paper. All authors have read and approved the final version of the manuscript.

## Pre-publication history

The pre-publication history for this paper can be accessed here:



## References

[B1] Giacinti C, Giordano A (2006). RB and cell cycle progression. Oncogene.

[B2] Sun A, Bagella L, Tutton S, Romano G, Giordano A (2007). From G0 to S phase: a view of the roles played by the retinoblastoma (Rb) family members in the Rb-E2F pathway. J Cell Biochem.

[B3] Cobrinik D, Lee MH, Hannon G, Mulligan G, Bronson RT, Dyson N, Harlow E, Beach D, Weinberg RA, Jacks T (1996). Shared role of the pRB-related p130 and p107 proteins in limb development. Genes Dev.

[B4] Lee MH, Williams BO, Mulligan G, Mukai S, Bronson RT, Dyson N, Harlow E, Jacks T (1996). Targeted disruption of p107: functional overlap between p107 and Rb. Genes Dev.

[B5] Hamel PA, Phillips RA, Muncaster M, Gallie BL (1993). Speculations on the roles of RB1 in tissue-specific differentiation, tumor initiation, and tumor progression. Faseb J.

[B6] Semczuk A, Jakowicki JA (2004). Alterations of pRb1-cyclin D1-cdk4/6-p16(INK4A) pathway in endometrial carcinogenesis. Cancer Lett.

[B7] Hocker TL, Singh MK, Tsao H (2008). Melanoma genetics and therapeutic approaches in the 21st century: moving from the benchside to the bedside. J Invest Dermatol.

[B8] Helmbold H, Deppert W, Bohn W (2006). Regulation of cellular senescence by Rb2/p130. Oncogene.

[B9] Baldi A, Boccia V, Claudio PP, De Luca A, Giordano A (1996). Genomic structure of the human retinoblastoma-related Rb2/p130 gene. Proc Natl Acad Sci USA.

[B10] Yeung RS, Bell DW, Testa JR, Mayol X, Baldi A, Grana X, Klinga-Levan K, Knudson AG, Giordano A (1993). The retinoblastoma-related gene, RB2, maps to human chromosome 16q12 and rat chromosome 19. Oncogene.

[B11] Chestukhin A, Litovchick L, Rudich K, DeCaprio JA (2002). Nucleocytoplasmic shuttling of p130/RBL2: novel regulatory mechanism. Mol Cell Biol.

[B12] Baldi A, Esposito V, De Luca A, Fu Y, Meoli I, Giordano GG, Caputi M, Baldi F, Giordano A (1997). Differential expression of Rb2/p130 and p107 in normal human tissues and in primary lung cancer. Clin Cancer Res.

[B13] Susini T, Massi D, Paglierani M, Masciullo V, Scambia G, Giordano A, Amunni G, Massi G, Taddei GL (2001). Expression of the retinoblastoma-related gene Rb2/p130 is downregulated in atypical endometrial hyperplasia and adenocarcinoma. Hum Pathol.

[B14] Lazzi S, Bellan C, De Falco G, Cinti C, Ferrari F, Nyongo A, Claudio PP, Tosi GM, Vatti R, Gloghini A (2002). Expression of RB2/p130 tumor-suppressor gene in AIDS-related non-Hodgkin's lymphomas: implications for disease pathogenesis. Hum Pathol.

[B15] Zamparelli A, Masciullo V, Bovicelli A, Santini D, Ferrandina G, Minimo C, Terzano P, Costa S, Cinti C, Ceccarelli C (2001). Expression of cell-cycle-associated proteins pRB2/p130 and p27kip in vulvar squamous cell carcinomas. Hum Pathol.

[B16] Russo G, Zamparelli A, Howard CM, Minimo C, Bellan C, Carillo G, Califano L, Leoncini L, Giordano A, Claudio PP (2005). Expression of cell cycle-regulated proteins pRB2/p130, p107, E2F4, p27, and pCNA in salivary gland tumors: prognostic and diagnostic implications. Clin Cancer Res.

[B17] Massaro-Giordano M, Baldi G, De Luca A, Baldi A, Giordano A (1999). Differential expression of the retinoblastoma gene family members in choroidal melanoma: prognostic significance. Clin Cancer Res.

[B18] Paggi MG, Giordano A (2001). Who is the boss in the retinoblastoma family? The point of view of Rb2/p130, the little brother. Cancer Res.

[B19] Santopietro R, Shabalova I, Petrovichev N, Kozachenko V, Zakharova T, Pajanidi J, Podistov J, Chemeris G, Sozaeva L, Lipova E (2006). Cell cycle regulators p105, p107, Rb2/p130, E2F4, p21CIP1/WAF1, cyclin A in predicting cervical intraepithelial neoplasia, high-risk human papillomavirus infections and their outcome in women screened in three new independent states of the former Soviet Union. Cancer Epidemiol Biomarkers Prev.

[B20] Sato T, Tanigami A, Yamakawa K, Akiyama F, Kasumi F, Sakamoto G, Nakamura Y (1990). Allelotype of breast cancer: cumulative allele losses promote tumor progression in primary breast cancer. Cancer Res.

[B21] Carter BS, Ewing CM, Ward WS, Treiger BF, Aalders TW, Schalken JA, Epstein JI, Isaacs WB (1990). Allelic loss of chromosomes 16q and 10q in human prostate cancer. Proc Natl Acad Sci USA.

[B22] Fujimori M, Tokino T, Hino O, Kitagawa T, Imamura T, Okamoto E, Mitsunobu M, Ishikawa T, Nakagama H, Harada H (1991). Allelotype study of primary hepatocellular carcinoma. Cancer Res.

[B23] Sato T, Saito H, Morita R, Koi S, Lee JH, Nakamura Y (1991). Allelotype of human ovarian cancer. Cancer Res.

[B24] Helin K, Holm K, Niebuhr A, Eiberg H, Tommerup N, Hougaard S, Poulsen HS, Spang-Thomsen M, Norgaard P (1997). Loss of the retinoblastoma protein-related p130 protein in small cell lung carcinoma. Proc Natl Acad Sci USA.

[B25] Claudio PP, Howard CM, Fu Y, Cinti C, Califano L, Micheli P, Mercer EW, Caputi M, Giordano A (2000). Mutations in the retinoblastoma-related gene RB2/p130 in primary nasopharyngeal carcinoma. Cancer Res.

[B26] Cinti C, Leoncini L, Nyongo A, Ferrari F, Lazzi S, Bellan C, Vatti R, Zamparelli A, Cevenini G, Tosi GM (2000). Genetic alterations of the retinoblastoma-related gene RB2/p130 identify different pathogenetic mechanisms in and among Burkitt's lymphoma subtypes. Am J Pathol.

[B27] Klumb CE, Magluta EP, Rezende LM, Apa AG, Alonso JF, Maia RC (2007). Retinoblastoma-related geneRb2/p130 are rarely mutated in Burkitt's lymphoma from Brazil. Am J Hematol.

[B28] Wright DH, Templeton AC (1973). Lymphoreticular Neoplasms. Tumors in a Tropical Country: a Survey of Uganda 1964-1968.

[B29] Ogwang MD, Bhatia K, Biggar RJ, Mbulaiteye SM (2008). Incidence and geographic distribution of endemic Burkitt lymphoma in northern Uganda revisited. Int J Cancer.

[B30] Orem J, Mbidde EK, Lambert B, de Sanjose S, Weiderpass E (2007). Burkitt's lymphoma in Africa, a review of the epidemiology and etiology. Afr Health Sci.

[B31] Tumwine LK, Campidelli C, Righi S, Neda S, Byarugaba W, Pileri SA (2008). B-cell non-Hodgkin lymphomas in Uganda: an immunohistochemical appraisal on tissue microarray. Hum Pathol.

[B32] Leoncini L, Bellan C, Cossu A, Claudio PP, Lazzi S, Cinti C, Cevenini G, Megha T, Laurini L, Luzi P (1999). Retinoblastoma-related p107 and pRb2/p130 proteins in malignant lymphomas: distinct mechanisms of cell growth control. Clin Cancer Res.

[B33] Kiviniemi M, Sauroja I, Rajamaki A, Punnonen K, Soderstrom KO, Salminen E (2000). Cell cycle regulators p27 and pRb in lymphomas - correlation with histology and proliferative activity. Br J Cancer.

[B34] Del Valle L, Baehring J, Lorenzana C, Giordano A, Khalili K, Croul S (2001). Expression of a human polyomavirus oncoprotein and tumour suppressor proteins in medulloblastomas. Mol Pathol.

[B35] Claudio PP, Zamparelli A, Garcia FU, Claudio L, Ammirati G, Farina A, Bovicelli A, Russo G, Giordano GG, McGinnis DE (2002). Expression of cell-cycle-regulated proteins pRb2/p130, p107, p27(kip1), p53, mdm-2, and Ki-67 (MIB-1) in prostatic gland adenocarcinoma. Clin Cancer Res.

[B36] Huynh H (2004). Overexpression of tumour suppressor retinoblastoma 2 protein (pRb2/p130) in hepatocellular carcinoma. Carcinogenesis.

[B37] Cinti C, Claudio PP, Howard CM, Neri LM, Fu Y, Leoncini L, Tosi GM, Maraldi NM, Giordano A (2000). Genetic alterations disrupting the nuclear localization of the retinoblastoma-related gene RB2/p130 in human tumor cell lines and primary tumors. Cancer Res.

